# Room temperature crystal field splitting of curium resolved by circularly polarized luminescence spectroscopy†

**DOI:** 10.1039/d4sc07594c

**Published:** 2025-02-06

**Authors:** Joshua J. Woods, Appie Peterson, Joseph A. Adewuyi, Rachael Lai, Jennifer N. Wacker, Rebecca J. Abergel, Gaël Ung

**Affiliations:** a Chemical Sciences Division, Lawrence Berkeley National Laboratory Berkeley California 94720 USA; b Department of Chemistry, University of Connecticut Storrs Connecticut 06269 USA gael.ung@uconn.edu; c Department of Nuclear Engineering and Department of Chemistry, University of California, Berkeley Berkeley CA 94720 USA abergel@berkeley.edu

## Abstract

Coordination of Cm(iii) with a chiral decadentate ligand *N*,*N*,*N*′,*N*′-tetrakis[(6-carboxypyridin-2-yl)methyl]-1,2-diaminocyclohexane (tpadac) generated complexes with strong luminescence allowing for the unprecedented measurement of well-resolved Cm(iii) circularly polarized luminescence spectra. Quantitative resolution of the electronic structure of the [Cm(tpadac)][K] complexes was achieved at room temperature, highlighting the strength of the combination of luminescence and circularly polarized luminescence spectroscopies to unravel the fundamental electronic structure of Cm(iii). These results are a clear demonstration that these spectroscopies are powerful yet simple tools for the fundamental understanding of electronic structure, which opens the door to future investigations of other Cm(iii) complexes in geometries relevant to nuclear applications, and even other 5f-elements.

## Abstract 2

Using a chiral *C*_2_-symmetrical ligand with picolinate antennae, a chiral emissive curium molecular complex was obtained. A well-resolved CPL spectrum was obtained allowing for deconvolution of the fundamental energy level of Cm^3+^ at room temperature.

## Graphical abstract

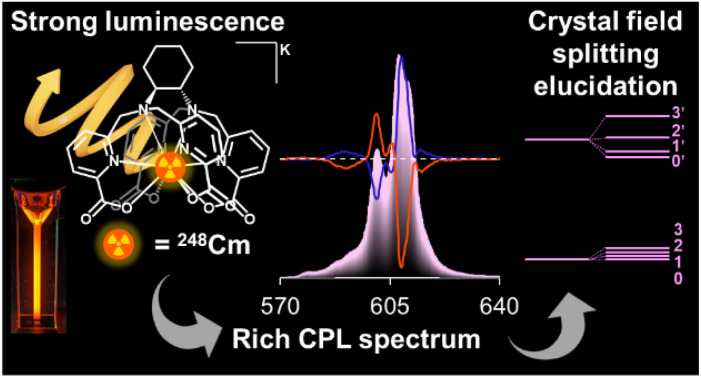

## Introduction

The current revitalization of the civil nuclear industry coupled with renewed geopolitical tensions underscores the need for a fundamental understanding of the chemistry of the transplutonium elements. In the event of accidental or otherwise uncontrolled environmental release of nuclear material, reclamation of small amounts of disseminated radioactive isotopes and identification of their origin would be critical.^1^ The reclamation of radioactive metals can be aided by a fundamental understanding of their coordination chemistry to design better chelates and extractants.^2^ Additionally, identification of the elements present in radioactive materials can be facilitated by the introduction of “intentional forensic tags”. The development of such tags requires precise fundamental knowledge of the electronic structure of the elements involved.^3^

Curium (Cm) is an important element to study not only for its presence as a by-product in spent nuclear fuel,^4^ but also for its unique photophysical properties that could be exploited for nuclear forensics applications. Like its isoelectronic lanthanide counterpart, Gd(iii), Cm(iii) features a half-filled f-shell with seven unpaired electrons. Due to spin–orbit coupling, the energy gap between the ground state and excited states of Cm(iii) (∼16 800 cm^−1^)^5^ is significantly smaller than that of Gd(iii) (30 000 cm^−1^). This unique property results in strong red-orange luminescence centered around 595 nm, which makes Cm(iii) particularly suitable for spectroscopic detection at nanomolar concentrations.^6^ The photophysical properties of Cm(iii) are highly sensitive to the coordination environment and electronic structure of the metal ion. In this context, luminescence from Cm(iii) has been exploited to determine the hydration number of the aqua ion.^7^ Both the ground and excited states of Cm(iii) are highly sensitive to the coordination environment around the metal center. The nature of the surrounding atoms can cause these states to exhibit crystal field splitting, which can provide important information related to the electronic structure of this ion. The magnitude of this splitting is highly dependent on the nature of the coordinating ligand and the symmetry of the complex.^8^ Crystal field splitting of the ground and excited states of Cm(iii) is often small and difficult to quantify in solution, especially at room temperature.^9^ As such, researchers have resorted to performing these analyses in the solid state or at cryogenic temperatures.^10^ Such measurements can be experimentally challenging, especially when involving rigorous engineering controls for safely handling highly radioactive samples in breakable vessels. Additionally, data obtained from cryogenic measurements may not be representative of the behavior of the element in environments relevant to nuclear applications.

Due to their expected chemical and electronic similarities, 5f-elements and 4f-elements should present similar spectroscopic behavior. Circularly polarized luminescence (CPL) spectroscopy is the measure of the preferential emission of right- or left-handed circularly polarized light. Because of the core-like nature of 4f orbitals, the splitting between energy levels of 4f-elements is larger through spin–orbit coupling than through crystal field splitting. In 4f-elements, each luminescent transition between spin–orbit coupling term levels is associated with a specific transition type, resulting in different relative CPL strengths.^11^ More importantly, CPL spectroscopy allows for better distinction of the individual components resulting from crystal field splitting, though these transitions' selection rules are not well understood. Since the strength of CPL spectroscopy to resolve fundamental electronic structures has been demonstrated with 4f-elements,^12^ we were motivated to use CPL spectroscopy as a tool to further understand the electronic structure of 5f-elements, specifically transplutonium elements. Since CPL typically requires relatively strong luminescence signals to provide resolved signals, curium was the 5f-element of choice to use as a proof-of-concept. We show below that by employing an appropriate ligand, well-resolved CPL spectra of a transplutonium element could be observed. More importantly, the quality of the data allowed for the deconvolution of the energy levels providing experimental mapping of the fundamental electronic structure of a Cm(iii) coordination complex in aqueous solution at room temperature.

## Results and discussion

### Synthesis

**Fig. 1:**
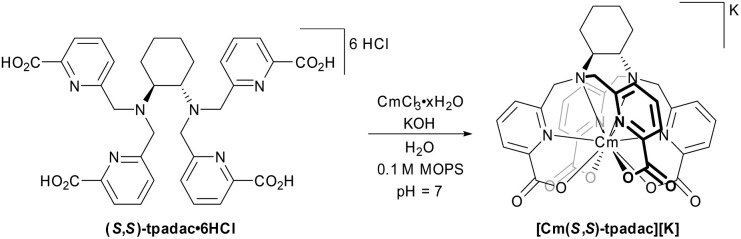
Synthesis of [Cm(*S*,*S*)-tpadac][K], the other enantiomer was synthesized analogously using (*R*,*R*)-tpadac. MOPS: 3-(*N*-morpholino)propanesulfonic acid.

We have recently shown that the use of *N*,*N*,*N*′,*N*′-tetrakis[(6-carboxypyridin-2-yl)methyl]-1,2-diaminocyclo-hexane (tpadac) (Fig. 1), a decadentate ligand containing a *trans*-1,2-diaminocyclohexane backbone and picolinate chelating groups, to generate complexes of trivalent lanthanides that were both highly luminescent in water and CPL active.^13^ The combination of having a well-characterized model system, strong luminescence, ability to generate CPL, and the potential for a strong ligand field prompted us to examine the coordination chemistry and photophysical properties of this chelator with curium.

To limit exposure to the highly radioactive curium isotope (Cm-248: *t*_1/2_ = 348 000 years, decaying by alpha decay and spontaneous fission), the synthetic procedure was adapted to work on a microgram scale and was designed to limit the number of hands-on operations. Briefly, a buffered solution of ligand was added to a solid residue of CmCl_3_·*x*H_2_O and the solution was allowed to equilibrate before being directly transferred to a cuvette for spectroscopic measurements (see ESI for details†). The procedure was validated using terbium as a surrogate and the spectroscopic data obtained were identical to those reported in the literature (see ESI for details†).^13^

### Optical properties

**Fig. 2:**
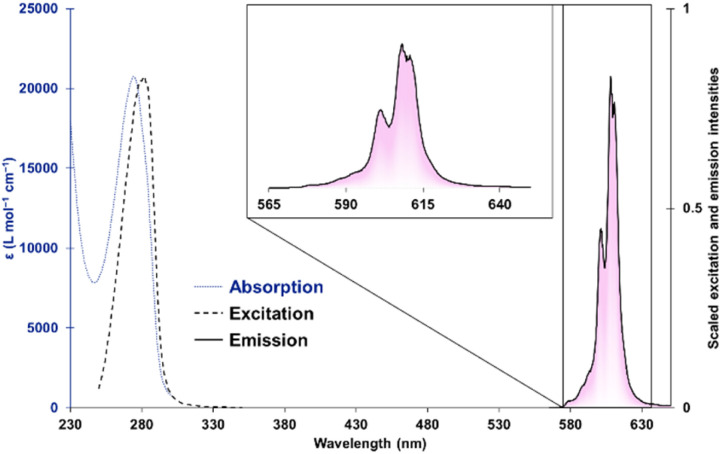
Absorption (dotted), excitation (dashed), emission (solid) spectra of [Cm(*S*,*S*)-tpadac][K] (4.7 μM) in 0.1 M MOPS buffer (pH 7). Insert: zoom in of the emission spectrum. MOPS: 3-(*N*-morpholino)propanesulfonic acid.

The absorption spectrum of [Cm(*S*,*S*)-tpadac][K] is consistent with all [Ln(*S*,*S*)-tpadac][K] complexes, with a single absorption feature centered at 270 nm and attributed to the picolinate π–π* transition (Fig. 2).^14^ Upon excitation at 280 nm, a sharp luminescence feature was observed between 570 and 630 nm, consistent with picolinate-sensitized emission from the ^6^D_7/2_/^6^P_7/2_ → ^8^S_7/2_ transition of Cm(iii).^10*a*,15^ Visual inspection of the luminescence spectrum clearly reveals the contribution of multiple overlapping features (see chiroptical analyses below). The excitation spectrum measured at the peak maximum (609 nm) is also consistent with what was previously observed with all [Ln(*S*,*S*)-tpadac][K] complexes.^13^ The luminescence lifetime of [Cm(*S*,*S*)-tpadac][K] was measured to be 0.723 ms and the decay was fitted to a monoexponential, consistent with the presence of a single emissive species in solution. The quantum yield, which was measured using an integrating sphere, was determined to be 13%. This is lower than that of the analogous terbium complex (47%) and slightly higher than the europium complex (6.5%), which is consistent with the expected positions of the emissive levels of Tb, Cm, and Eu (20 500, 17 100, and 17 300 cm^−1^, respectively)^16^ relative to the triplet state of the ligand (22 272 cm^−1^).^17^ Additionally, to examine the possibility of luminescence quenching by inner-sphere water molecules, we measured the lifetimes in various H_2_O/D_2_O mixtures to determine the number of coordinated water molecules (*q*) through the fit initially described by Kimura and Choppin.^7*d*^ Our experiments suggest a *q* value of −0.2 ± 0.01 (with an equation error estimated at ±0.5 water molecules^7*d*^). These results are consistent with no bound water molecules (see ESI†). Overall, all the data collected through optical spectroscopy are consistent with a similar coordination environment around curium when compared with the lanthanides. We thus proceeded with chiroptical measurements.

### Chiroptical properties

**Fig. 3:**
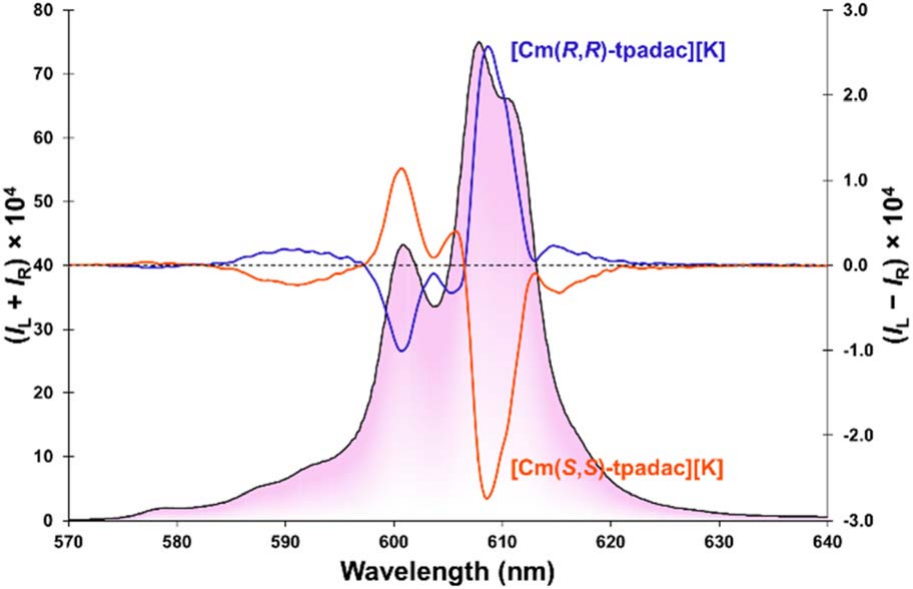
CPL spectra of [Cm(*S*,*S*)-tpadac][K] (orange) and [Cm(*R*,*R*)-tpadac][K] (blue) in solution in 0.1 M MOPS buffer (4.7 μM). The total luminescence is traced in the background. Excitation: 280 nm, bandpass: 0.5 nm. MOPS: 3-(*N*-morpholino)propanesulfonic acid.

Upon excitation at 280 nm, the [Cm(*S*,S)-tpadac][K] complex displayed circularly polarized luminescence (Fig. 3). Thanks to the relatively strong quantum yield of the complex, the CPL spectrum of [Cm(*S*,*S*)-tpadac][K] is well resolved and at least seven individual luminescent components can be clearly observed. This contrasts starkly with previously published curium CPL spectra, which are noisy and featureless.^18^ A maximum dissymmetry factor was observed at 609 nm at −0.07 although another luminescent contribution at 589 nm displays a close dissymmetry factor at −0.06. These values may be slightly larger than those of previously reported CPL-active curium complexes (|*g*_lum_|∼0.01).^18^ However, it has been shown that CPL metrics can vary greatly if the measurements were not performed under identical conditions (different bandpass),^12,19^ it is thus safer to consider the metrics of [Cm(*S*,*S*)-tpadac][K] to be of the same order of magnitude (Table 1). The spectrum of [Cm(*R*,*R*)-tpadac][K] was the expected mirror-image. The CPL brightness (*B*_CPL_) was calculated to be 165 L mol^−1^ cm^−1^ using the simpler equation introduced in 2020,^20^ we also calculated the ^An^*B*_CPL_ (83.5 L mol^−1^ cm^−1^) utilizing an equation we developed that accounts for the different sign contribution of the m_*J*_ states.^21*a*^

**Table 1:** Summarized photophysical data for [Cm(*S*,*S*)/(*R*,*R*)-tpadac][K]

*Φ*	*τ* (ms)	Max *g*_lum_	*B*_CPL_ (L mol^−1^ cm^−1^)	^An^ *B*_CPL_^21^ (L mol^−1^ cm^−1^)
0.13	0.723	±0.07	165	83.5

**Fig. 4:**
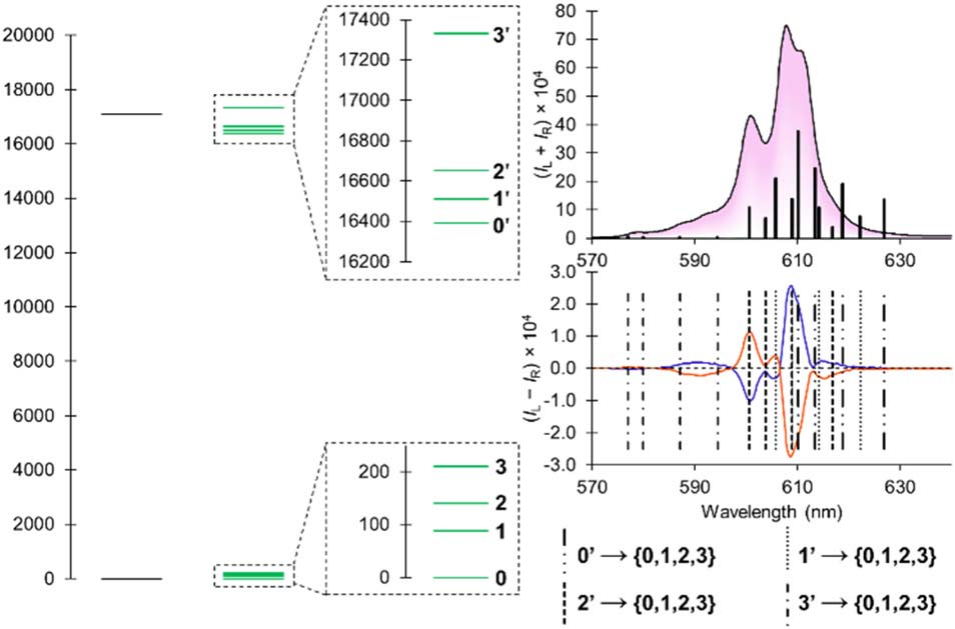
(Left) Fundamental electronic structure of [Cm(*S*,*S*)-tpadac][K]; (top right) emission spectrum overlayed with predicted transitions as vertical black bars (the intensity is representative of a statistical Boltzmann distribution); (bottom right) CPL spectrum overlayed with predicted transitions as vertical dashed lines.

With the availability of well-resolved luminescence and circularly polarized luminescence spectra, we hypothesized the data obtained was sufficient to resolve the electronic structure of the [Cm(*S*,*S*)-tpadac][K] complex at room temperature. For Cm(iii), the luminescent transition arises from a mixed ^6^D_7/2_/^6^P_7/2_ excited state to a ground ^8^S_7/2_ state.^22^ In *C*_2_-symmetry, both levels can theoretically be split by crystal field into a maximum of four individual m_*J*_ states, yielding sixteen possible luminescent transitions.^23^ Using both the luminescence and CPL spectra (Fig. 4), we were able to estimate the position of those sixteen transitions (see ESI for details†). This allowed the experimental mapping of the individual m_*J*_ states of the excited state (split into 4 states at 16 390, 16 510, 16 650, and 17 331 cm^−1^) and ground state (split into 4 states at 0, 88, 140, and 210 cm^−1^). The magnitude of the splitting is significantly greater than that of the free ion in a LuPO_4_ lattice (∼1–20 cm^−1^)^24^ and highlights the strong effect of ligand chelation on the splitting of these states. The fitted luminescence spectrum, using a theoretical statistical Boltzmann population of the states, is in reasonable agreement with the experimental data (black bars in Fig. 4 and S8† for Lorentzian fit). By fitting the CPL spectrum (see Fig. S9†), we also determined the sign for each transition between crystal field splitting levels (see Table S1†). This information could prove crucial for a broader study of the selection rules between crystal field splitting levels of 5f-elements. Lower temperature luminescence and CPL measurement could provide better precision, however, those experiments are logistically challenging as described above.

## Conclusions

By using a ligand designed to enable strong luminescence, circularly polarized luminescence, and strong crystal field splitting, we have mapped the electronic structure of both the excited and ground states of a molecular complex of Cm(iii) at room temperature. This unprecedented achievement was only possible thanks to the combination of luminescence and circularly polarized luminescence studies. Our study demonstrates that the electronic structure of curium and likely other luminescent actinides can be established at room temperature using readily available spectroscopic techniques, thus providing a clear path towards a deeper fundamental understanding of these rare, but critical elements in environmentally relevant conditions. Additionally, the magnitude of the crystal field splitting observed is significant enough to hypothesize that thorough structure/function studies will allow us and others to correlate ligand field, covalency, and electronic structures in a more precise and quantitative manner.

## Data availability

The data supporting this article have been included as part of the ESI.†

## Author contributions

Conceptualization: JJW, RJA, GU; investigation: JJW, AP, JAA, RL, JNW, GU; data curation: JJW, AP, JNW, GU; writing – original draft: JJW, GU; writing – reviewing & editing: JJW, AP, JAA, JNW, RJA, GU; funding acquisition: GU, RJA.

## Conflicts of interest

There are no conflicts to declare.

## Supplementary Material

SC-OLF-D4SC07594C-s001
