# Supplementary material for: Room temperature crystal field splitting of curium resolved by circularly polarized luminescence spectroscopy
Source: Chem Sci. 2025 Feb 6;16(11):4815–20. doi: 10.1039/d4sc07594c (PMC11815341; doi:10.1039/d4sc07594c)
Supplement: SC-016-D4SC07594C-s001 [file SC-016-D4SC07594C-s001.pdf]

Supplementary Information (SI) for Chemical Science.
This journal is © The Royal Society of Chemistry 2025

Supporting Information

Room Temperature Crystal Field Splitting of Curium Resolved by Circularly Polarized Luminescence Spectroscopy

Joshua J. Woods,[a] *Appie Peterson,*[a] *Joseph A. Adewuyi,*[b] *Rachael Lai,*[b] *Jennifer N. Wacker,*[a] *Rebecca J. Abergel,*[a,c,*] *Gaël Ung*[a,b,*]

[a] Chemical Sciences Division, Lawrence Berkeley National Laboratory, Berkeley, CA 94720, United States.

[b] Department of Chemistry, University of Connecticut, Storrs, CT 06269, United States.

[c] Department of Nuclear Engineering and Department of Chemistry, University of California, Berkeley, Berkeley, CA 94720, United States.

Table of Contents

General Materials and Methods 3
Engineering control and safety precautions 3
Materials and chemicals 3
Spectroscopy Measurements 3
Isotope reprocessing and purification 4
Synthesis and Characterizations 4
Luminescence Scale Validation with Terbium 4
Synthesis of [Cm(S,S)-tpadac][K] 4
Supplemental Figures 5
Determination of the crystal field splitting 8
References 10

General Materials and Methods

Engineering control and safety precautions

Caution! Curium isotopes (^{248}Cm: $t_{1/2}$ = 3.48 x 10^5 y, ^{246}Cm: $t_{1/2}$ = 4706 y, ^{244}Cm: $t_{1/2}$ = 18.11 y)[1] and their daughters present serious health threats due to spontaneous fission and the emission of α-, β-, and γ-radiation. **Note**–the curium stock used in this work was primarily ^{248}Cm by mass (^{248}Cm: 97%, ^{246}Cm: 3%, ^{244}Cm: <0.001%), while the majority of the activity was attributable to ^{246}Cm (^{248}Cm: 33%, ^{246}Cm: 65%, ^{244}Cm: 1.9%). All radioactive materials were handled in a facility–the Heavy Element Research Laboratory (HERL) at Lawrence Berkeley National Laboratory (LBNL)–designed in accordance with appropriate safety protocols and engineering controls including high-efficiency particulate air (HEPA) filtered negative pressure gloveboxes and fume hoods, α- and β-counting portable survey meters, benchtop scalers, and a Canberra Sirius 5PAB hand-and-foot personal contamination monitoring station at the entrance/exit. Body and extremity personnel dosimetry were worn by all researchers in addition to standard laboratory personal protective equipment. All manipulations involving dispersible radioactive materials were performed in a negative pressure glovebox.

Materials and chemicals

<u>Materials:</u>

All manipulations of solutions containing radioisotopes were performed using low retention pipette tips (VWR International).

<u>Chemicals:</u>

All acids were of Optima grade (Fisher Scientific) unless otherwise noted. Aqueous solutions were prepared in ultrapure water (18 MΩ·cm), which was purified using a ZMQ56V001 Milli-Q water purification system (Millipore Inc.).

The tpadac ligands were prepared according to the previously reported procedure.[2]

<u>Fluorescence cuvettes:</u>

Starna Cells (18F-Q-10-GL14-C) with a screw cap.

Firefly Cells (46FLUV10X2) with a custom modification to fit the integrating sphere.

Spectroscopy Measurements

Absorption spectra were acquired at room temperature on a Cary 6000i UV-vis-NIR spectrometer (Agilent Technologies Inc.) controlled with Cary WinUV software.

Excitation, emission, and lifetime measurements were acquired at room temperature on a PicoQuant FluoTime300 spectrometer controlled with EasyTau 2 software.

Electronic circular dichroism (ECD) spectra were acquired at room temperature on a Jasco J-815 spectropolarimeter controlled using Spectra Manager II software. The data presented is an average of 3 scans with a scan rate of 50 nm/min and a digital integration time of 2 s.

Circularly Polarized Luminescence (CPL) spectra were acquired at room temperature on an Edinburgh FLS980 spectrometer fitted with a circularly polarized luminescence accessory (Edinburgh Instruments Ltd.) controlled with the F980 CPL application. The data presented is an average of 30 scans. Bandpass 2 nm. Integration time 0.3 s per 0.2 nm.

Liquid scintillation counting (LSC) was performed using a Perkin Elmer Tri-Carb 4910TR liquid scintillation counter controlled with QuantaSmart software (Perkin Elmer, Inc.). Samples were prepared by diluting 3 µL of metal solution into 5 mL of Ultima Gold liquid scintillation cocktail (Perkin Elmer, Inc.) and shaking prior to counting. Samples were counted for 10 min.

Isotope reprocessing and purification

The purified stock solution of curium utilized was obtained from a previous reprocessing campaign described in Peterson et al.[3]

Synthesis and Characterizations

Luminescence Scale Validation with Terbium

Reagent preparation:

A 0.1 M MOPS buffer solution was prepared and brought to pH = 7 with KOH.

A solution of *S*,*S*-*N*,*N*,*N′*,*N′*-tetrakis[(6-carboxypyridin-2-yl)methyl]-1,2-diaminocyclohexane (abbreviated *S*,*S*-tpadac) was prepared as a 0.143 mmol/L solution in 0.1 M MOPS buffer.

Procedure:

In air, in a 4 mL vial was added 100 µL of $TbCl_3{\cdot}6H_2O$ stock in 4M HCl (15 µg Tb^{3+} ,0.1 µmol Tb^{3+}). The solution was brought to a solid residue by evaporation facilitated by an air stream. 700 µL of the *S*,*S*-tpadac solution in MOPS was added and the suspension was swirled until a homogeneous solution was obtained. The vial was capped with a PTFE lined screw cap (VWR) and let sit at ambient temperature for at least 12 hours to equilibrate. The solution was transferred to a fluorescence cuvette. The enantiomer synthesis and characterization was done analogously. The luminescence spectrum and CPL spectra obtained are shown in Figure S1 and S2).

Synthesis of [Cm(S,S)-tpadac][K]

Reagent preparation:

A 0.1 M MOPS buffer solution was prepared and brought to pH~7 with KOH

A solution of *S*,*S*-*N*,*N*,*N′*,*N′*-tetrakis[(6-carboxypyridin-2-yl)methyl]-1,2-diaminocyclohexane (abbreviated *S*,*S*-tpadac) was prepared as a 0.143 mmol/L solution in 0.1 M MOPS buffer.

A solution of *R*,*R*-*N*,*N*,*N′*,*N′*-tetrakis[(6-carboxypyridin-2-yl)methyl]-1,2-diaminocyclohexane (abbreviated *R*,*R*-tpadac) was prepared as a 0.143 mmol/L solution in 0.1 M MOPS buffer.

Procedure:

In air, in a 4 mL vial was added 100 µL of Cm(III) stock in 4M HCl (20 µg Cm^{3+} ,0.1 µmol Cm^{3+}). The solution was brought to a solid residue by evaporation facilitated by an air stream. 700 µL of the *S*,*S*-tpadac solution in MOPS was added and the suspension was swirled until a homogeneous solution was obtained. The vial was capped with a PTFE lined screw cap (VWR) and let sit at ambient temperature for at least 12 hours to equilibrate. The solution was transferred to a fluorescence cuvette.
The enantiomer synthesis and characterization was done analogously using the *R*,*R*-tpadac solution.

Supplemental Figures

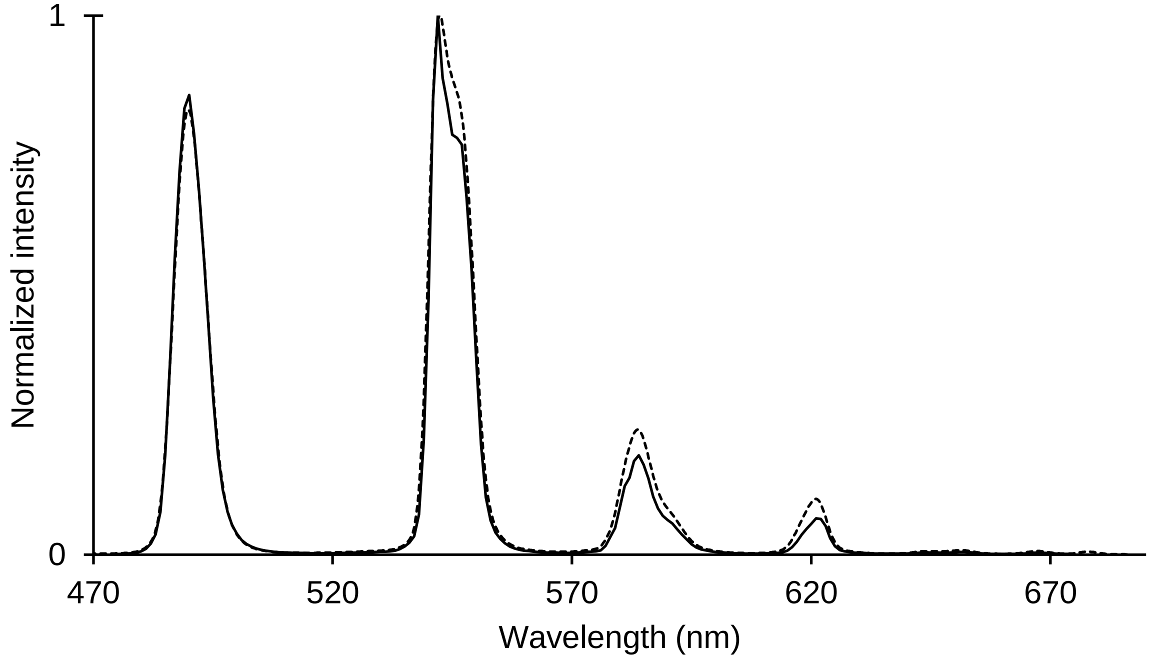

Figure S 1: Overlay of the normalized luminescence spectra of [Tb(*S,S*)-tpadac][K] prepared from the validation procedure (solid line) compared to the published spectrum of [Tb(*S,S*)-tpadac][K] (dashed line). Spectra were obtained with an excitation wavelength of 280 nm.

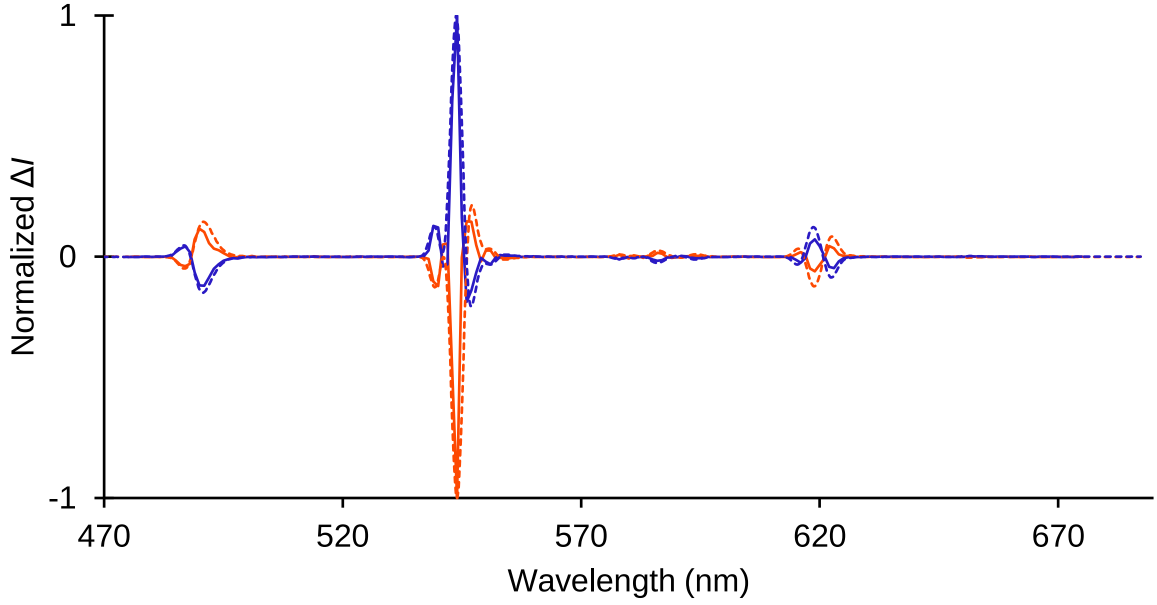

Figure S 2: Overlay of the normalized CPL spectra of [Tb(*S,S*)-tpadac][K] prepared from the validation procedure (solid orange) compared to the published spectrum of [Tb(*S,S*)-tpadac][K] (dashed orange). The enantiomeric traces for [Tb(*R,R*)-tpadac][K] are shown in solid and dashed blue. All spectra were obtained with an excitation wavelength of 280 nm.

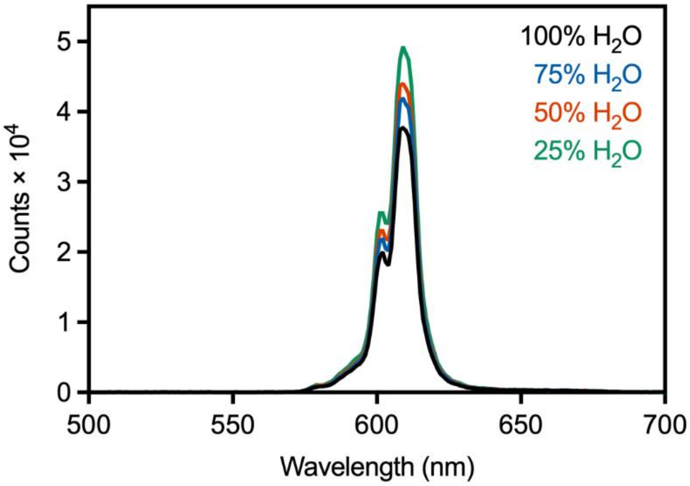

Figure S 3: Emission spectra of [Cm(*S*,*S*)-tpadac][K] (4.7 μM) in 0.1 M MOPS (pH 7) in 0.1 M MOPS (pH 7) buffer with varying H_2O:D_2O ratios. All spectra were obtained using an excitation wavelength of 275 nm.

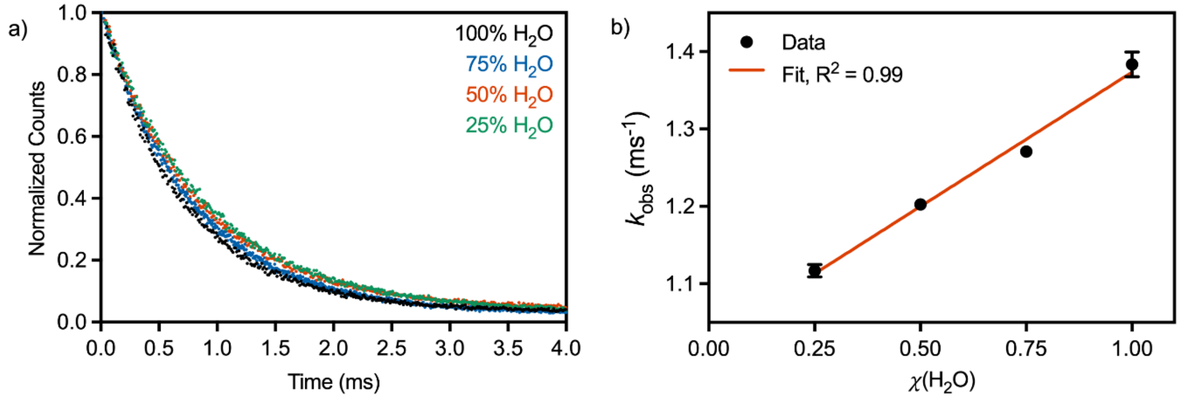

Figure S 4: (a) Representative decay curves for [Cm(*S*,*S*)-tpadac][K] (4.7 μM) in 0.1 M MOPS (pH 7) buffer with varying H_2O:D_2O ratios excited at 275 nm and measured at 609 nm (b) Fit of k_{obs} as a function of the molar fraction of H_2O. Results are reported as the average ± S.D. of three independent measurements. The k_{obs} in 100% D_2O was calculated from the linear dependency of k_{obs} on the molar fraction of H_2O to be 1.03 ± 0.01 ms^{-1} (τ = 0.97 ± 0.1 ms).

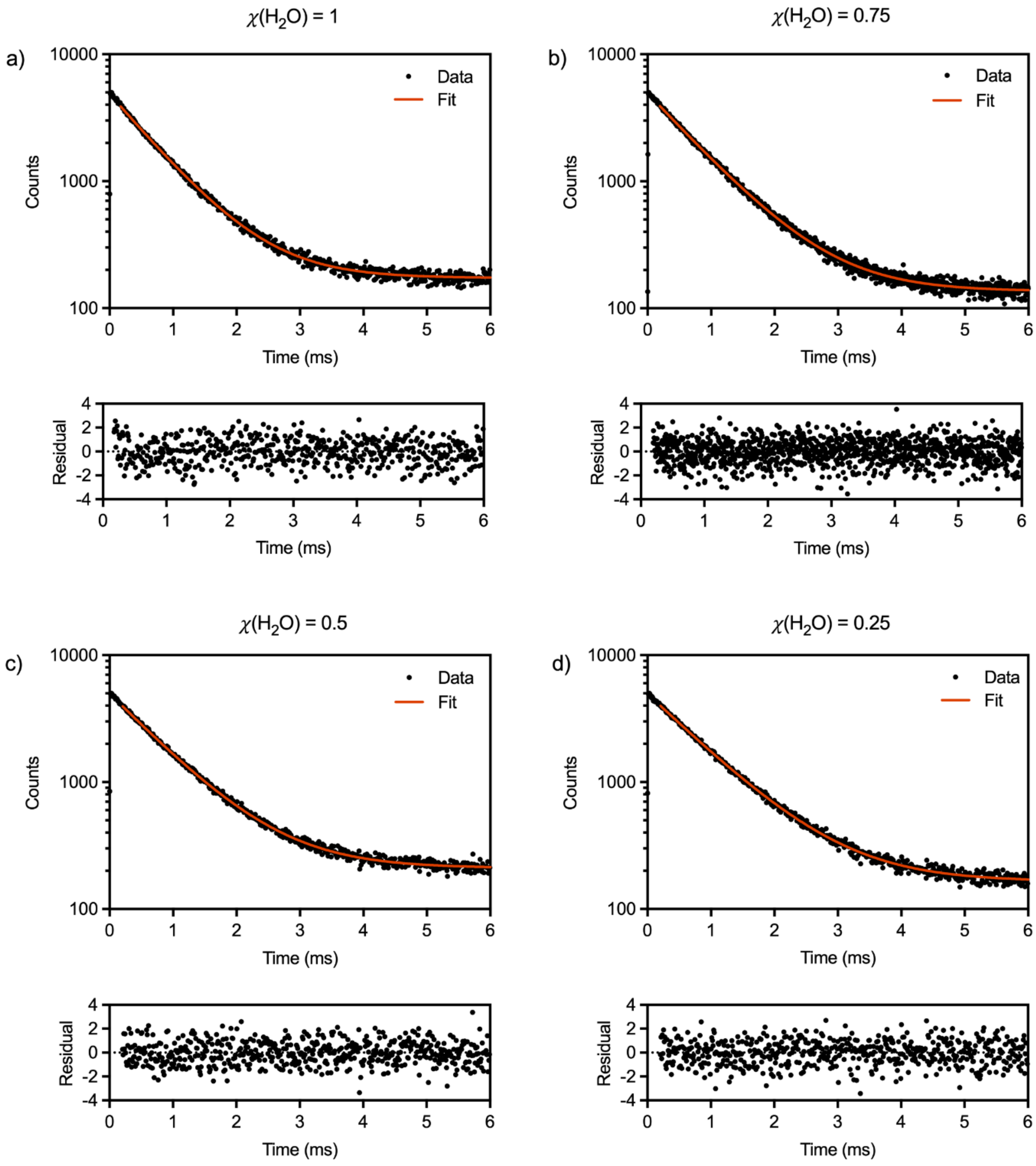

Figure S 5: Representative fits of the decay curve for [Cm(*S*,*S*)-tpadac][K] (4.7 μM) in 0.1 M MOPS (pH 7) buffer excited at 275 nm and measured at 609 nm in (a) 100:0 H_2O:D_2O (b) 75:25 H_2O:D_2O, (c) 50:50 H_2O:D_2O, and (d) 25:75 H_2O:D_2O.

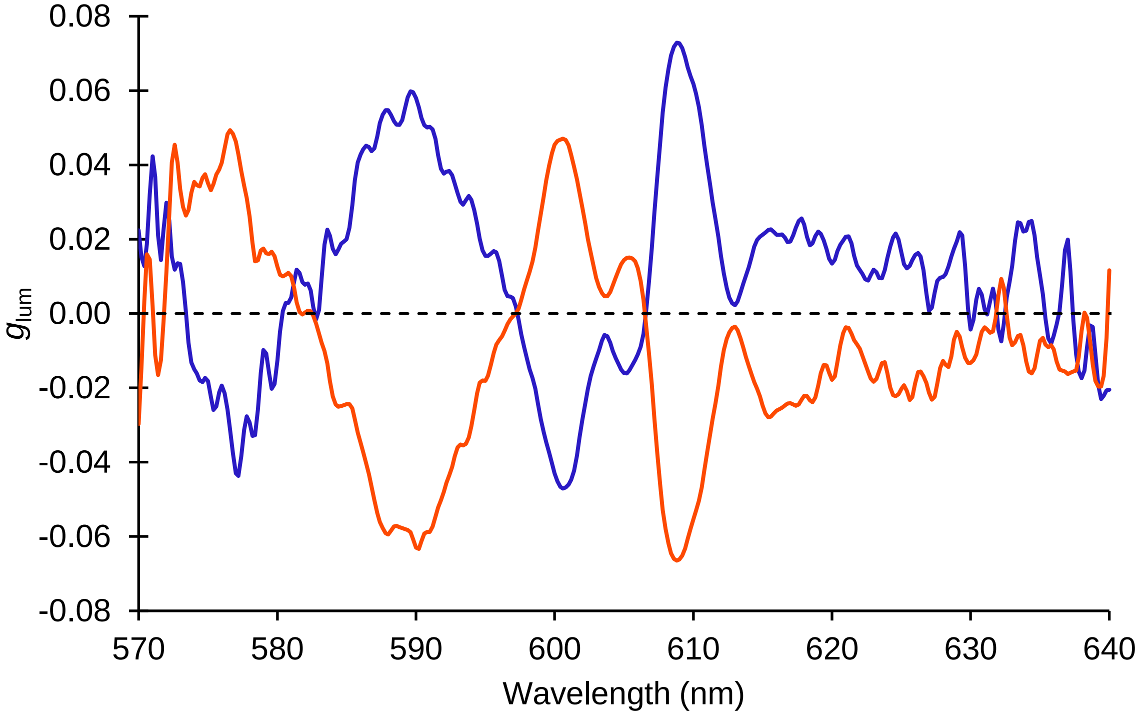

Figure S 6: g_{lum} vs. wavelength spectrum of [Cm(*R*,*R*)-tpadac][K] (blue) and [Cm(*S*,*S*)-tpadac][K] (orange). Spectra measured in 0.1 M MOPS buffer (200 µmol/L) at room temperature, 1 cm path cuvette. All spectra were obtained using an excitation wavelength of 280 nm.

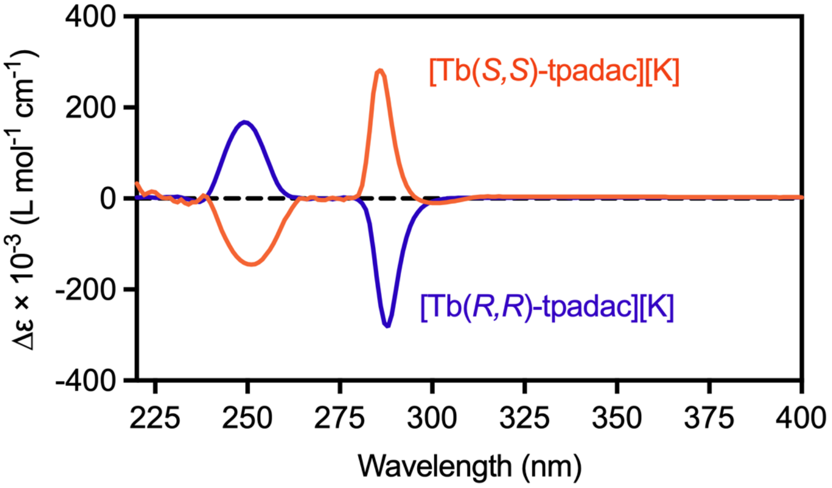

Figure S 7: Circular dichroism spectra of [Tb(*R*,*R*)-tpadac][K] (blue) and [Tb(*S*,*S*)-tpadac][K] (orange). Spectra measured in 0.1 M MOPS buffer (500 µmol/L, pH 7) at room temperature, 1 cm path cuvette.

Determination of the crystal field splitting

The crystal field splitting levels were determined by fitting the experimental luminescence and CPL spectra. First, relatively obvious transitions (e.g. 0'-0—high intensity—, 3'-0—highest energy)

were determined. Determination of the excited state (3', 2', 1') positions was done by examining peaks at higher energy from the 0'-0 transition. The ground state (3, 2, 1) positions were determined by seeking obvious transitions that were at lower energy from the respective transition to the ground state. "Obvious transition" refers to a maximum/minimum in both luminescence and CPL spectra. Once an initial guess for the positions was determined, probabilities of the population of the states were calculated using the Boltzmann equation utilizing the energy of the states obtained through fitting of the luminescence and CPL spectra, the Boltzmann constant, and the temperature (295 K). If the luminescence fit was not satisfactory, the initial guess was revised. The CPL fit was obtained by flipping some of the Lorentzian signs.

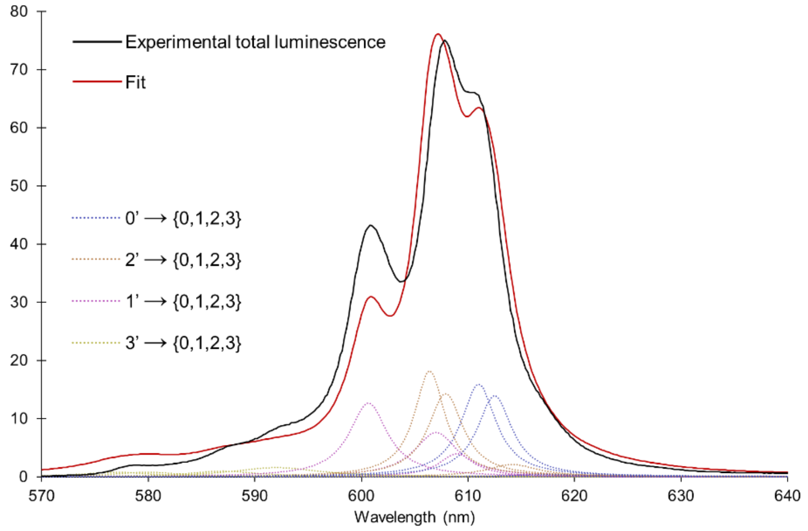

Figure S 8: Fitting of the luminescence spectrum utilizing Lorentzian functions. The position of the peaks and their area closely matches the lines shown in Figure 4 in the manuscript.

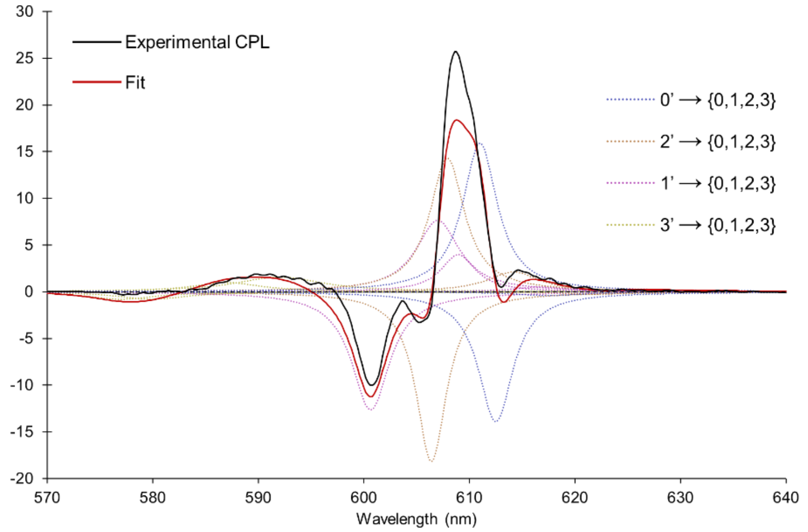

Figure S 9: Fitting of the CPL spectrum of [Cm(R,R)-tpadac][K] utilizing the Lorentzian functions utilized in Figure S7 with either positive or negative signs. The position of the peaks and their area closely matches the lines shown in Figure 4 in the manuscript.

Table S1**:** Sign of the transition between crystal field splitting levels for [Cm(*R*,*R*)-tpadac][K].

Transition	Sign	Transition	Sign	Transition	Sign	Transition	Sign
0'→0	positive	1'→0	negative	2'→0	negative	3'→0	negative
0'→1	negative	1'→1	positive	2'→1	positive	3'→1	negative
0'→2	negative	1'→2	positive	2'→2	positive	3'→2	positive
0'→3	positive	1'→3	negative	2'→3	positive	3'→3	positive

References

[1] *Nuclear Data Services - International Atomic Energy Agency*. https://www-nds.iaea.org/

[2] Adewuyi, J. A.; Schley, N. D.; Ung, G.* "Synthesis of bright water-soluble circularly polarized luminescence emitters as potential sensors" *Inorg. Chem. Front*. 2022**,** ***9***, 1474-1480. https://doi.org/10.1039/D1QI01398J

[3] Peterson, A.; Adewuyi, J. A.; Woods, J. J.; Lukens, W. W.; Abergel, R. J.; Ung, G. Consolidated Curium Reprocessing Procedure Inspires Molecular Design for Sensitized Curium Circularly Polarized Luminescence. *Inorg. Chem.* 2024, ***63***, 19752-19785. https://pubs.acs.org/doi/10.1021/acs.inorgchem.4c02976
